# Role of endothelin receptor signalling in squamous cell carcinoma

**DOI:** 10.3892/ijo.2011.1258

**Published:** 2011-11-10

**Authors:** SHUNSUKE ISHIMOTO, KOICHIRO WADA, NORIAKI TANAKA, TADASHI YAMANISHI, KOHJI ISHIHAMA, TOMONAO AIKAWA, MASAYA OKURA, ATSUSHI NAKAJIMA, MIKIHIKO KOGO, YOSHINORI KAMISAKI

**Affiliations:** 1 Department of Pharmacology, Graduate School of Dentistry, Osaka University, 1-8 Yamadaoka, Suita, Osaka 565-0871; 2 The First Department of Oral and Maxillofacial Surgery, Graduate School of Dentistry, Osaka University, 1-8 Yamadaoka, Suita, Osaka 565-0871; 3 Department of Gastroenterology, Yokohama City University School of Medicine, 3-9 Fuku-ura, Yokohama 236-0004, Japan

**Keywords:** squamous cell carcinoma, tongue, cellular proliferation, endothelin receptor, MAP kinase signaling pathway

## Abstract

Endothelin plays important roles in various physiological functions including vascular constriction. Recent studies reported that the endothelin receptors ET_A_ and ET_B_ are highly expressed in lung and skin tumor tissues. In contrast, there are few reports on endothelin signalling in the proliferation of head and neck cancer. We found that both ET_A_ and ET_B_ endothelin receptors were overexpressed in tumor cells of tongue cancer samples by immunohistochemistry. ET_A_ and ET_B_ were expressed in cultured lingual and esophageal squamous cell carcinoma (SCCs) cell lines. When both cultured cell lines were treated with an ET_A_ selective antagonist (BQ123) or an ET_B_ selective antagonist (BQ788), inhibition of cell growth was observed. Similar results were observed when SCCs were treated with specific siRNA for the suppression of ET_A_ or ET_B_. Furthermore, inhibition of the mitogen-activated protein (MAP) kinase pathway by the treatments with ET receptor antagonists and siRNA was also observed. These results indicate that endothelin signalling may, in part, play important roles in cell growth in SCCs through the MAP kinase pathway.

## Introduction

Endothelin (ET) plays important roles on various physiological functions including vascular constriction (1–4). ET family comprises three isoforms, ET-1, ET-2, and ET-3, that bind to two receptor subtypes, endothelin A (ET_A_) and endothelin B (ET_B_) receptors (1–4). Recent studies reported that ET_A_ and ET_B_ were highly expressed on lung, colon and skin cancers (5–7). In addition, several reports suggested that ET-1 plays important roles in tumorigenesis, tumor progression, and metastasis (8–10). Thus, the ET receptors and their signalling pathways may be a therapeutic target in cancer therapy (11). However, little is known about the role of ET signalling on tumor cell proliferation of oral squamous cell carcinoma (SCC).

Human SCC is major neoplasm in esophagus or oral cavity and the incidence has recently been increasing (12–14). The optimal treatment for early carcinoma of oral cavity is surgical operation. However, overall survival remains largely unchanged (12–14). In addition, the decrease in quality of life (QOL) after wide excision of tongue is also important issue for patients. Therefore, different therapies are required. In our previous studies, we investigated the whole genome analysis using DNA microarray to find the potential target genes involved in tumor cell growth, and reported the critical role of several important molecules on the cell growth of SCCs (15–19). According to the results of DNA microarray, we found increased expression of ET receptor mRNA in cell lines of oral SCCs and the alteration of expression level on SCC growth (15,18,19). Therefore, we have examined whether ET receptors may be expressed in primary oral SCC tissues, and whether ET receptor-signalling may play a critical role of SCC growth. Our results imply a potentially important and novel role of ET function on SCC growth, and suggest that ET receptor-signalling might be useful target in the therapy of SCCs.

## Materials and methods

### Tissue samples

All of clinical studies were approved by the Ethics Committee of Osaka University Dental Hospital. Twenty-three samples of squamous cell carcinoma (SCC) located in the tongue were obtained from surgical resection tissue specimens at Osaka University Dental Hospital after informed consent was obtained. The patients, who received no preoperative therapy including chemotherapy and irradiation therapy, were randomly selected (Table I). The age range was 33–92 years (average: 62.0±13.9 years, mean ± SD).

### Chemicals and antibodies

ET receptor specific antagonists, BQ123 for ET_A_ and BQ788 for ET_B_ were purchased from Sigma-Aldrich Japan (Tokyo, Japan). Anti-ET_A_ or ET_B_ polyclonal antibody was from Acris (Acris, Herford, Germany). Antibodies against Focal adhesion kinase (FAK), phosphorylated FAK, phosphorylated MEK1/2, p44/42 MAPK (pErk1/2) phosphorylated p44/42 MAPK and anti-rabbit IgG (HRP-linked) for secondary antibody are from Cell Signalling Technologies (Beverly, MA). Cisplatin was from Wako Pure Chemical Industries, Ltd. (Osaka, Japan).

### Immunohistochemical staining of ET_A_ and ET_B_

The expression of ET_A_ or ET_B_ in tissues was detected by anti-ET_A_ or ET_B_ specific polyclonal antibody using standard immunohistochemical techniques on formalin-fixed and paraffin-embedded continuous sections. Incubation with anti-ET_A_ or ET_B_ polyclonal antibody was performed at 4°C for 16 h, then the sections were washed out. After the application with secondary antibody, the Vectastain ABC kit (Vector Laboratories, Burlingame, CA) was used with a 3,3′-diaminobenzidine (DAB) substrate kit, according to the manufacturer’s instructions. The staining endpoint was determined when the standard tissue sections were constantly stained to the intensity as described previously (18,19).

The intensity of the immunohistochemical staining with anti-ET_A_ or ET_B_ antibody was evaluated by scoring the staining reaction in four groups: (−), none/weak; (+), weak/moderate; (++), moderate/strong, and (+++), very strong cytoplasmic staining intensity, respectively (18,19). To check the reproducibility of the evaluation system concerning the immunohistochemical staining for the ET_A_ and ET_B_ proteins, another oral surgeon and pathologist who were unaware of the original assessment re-evaluated the results of staining according to the system above. Tumor areas were confirmed by both of the pathologist and surgeon under the microscopy. Non-tumor areas were selected, the comparatively normal areas were separated away from the tumor areas, and confirmed by the pathologist.

### Cell culture

We used human oral SCC cell line (SAS) and human esophageal SCC cell line (KYSE70). SAS was established as tongue SCC and KYSE70 was established as esophageal SCC (15,16). SAS was maintained in DMEM containing 10% fetal bovine serum (FBS), and KYSE70 was maintained in DMEM containing 2% FBS at 37°C under 0.5% CO_2_. For cell growth experiment, cells were trypsinized and replated onto culture dishes (15–19).

### Cell survival assay using ET receptor antagonists

SCC cells were treated with ET receptor antagonists, BQ123 for ET_A_ and BQ788 for ET_B_ for 24 and 48 h in culture medium. Then, cell viability was measured 24 and 48 h after the treatment using Countess Automated Cell Counter (Invitrogen, Eugene, OR). The inhibition of cell growth was compared to vehicle-treated control.

### RNA interference approach

SAS and KYSE70 were trypsinized and resuspended in DMEM without FBS, and the cells were separated approximately 1×10^5^ cells for each dish. The ET_A_ and ET_B_-specific siRNA (Stealth siRNA) were purchased from Invitrogen Japan (Tokyo, Japan). The sequence of the sense strand of ET_A_-siRNA is 5′-UUUGAUGUGGCAUUGAGCAUACAGG-3′, and antisense is 5′-CCUGUAUGCUCAAUGCCACAUCAAA-3′, respectively. The sequence of the sense strand of ET_B_-siRNA is 5′-UAAUUCUACUCCAAGAAGCAACAGC-3′, and antisense is 5′-GCUGUUGCUUCUUGGAGUAGAAUUA-3′, respectively. For the transfection, ET_A_, ET_B_-siRNA (40 nM) or negative control (40 nM Stealth RNAi Negative Control Duplexes, Invitrogen Japan Inc.) solution was added to DMEM medium containing Lipofectamine RNAiMax (Invitrogen Japan) and allowed to incubate for 20 min at room temperature to create the transfection mixture. The transfection mixture was then added to the cells at the indicated final concentration of siRNA. Twenty-four hours after the transfection, the medium was changed to DMEM containing 10% FBS for SAS and 2% FBS for KYSE70. Then, viable cell number was measured 24 and 48 h after the medium change using Countess Automated Cell Counter. The cell growth was expressed as the percentage to that of vehicle control.

### Western blot analysis

Adherent or suspended cells were washed in PBS, and cell extracts were prepared by lysing cells in lysis buffer. The proteins were separated by electrophoresis using 10% SDS-PAGE, and transferred to nitrocellulose membrane (Millipore, Bedford, MA). Detection of proteins were performed by each polyclonal antibody and visualized by using the ECL detection kit (Amersham, London, UK) following the manufacturer’s suggested procedure.

### Combination of ET_A_, ET_B_-siRNA and anti-tumor drug

Com- bined treatment of ET_A_, ET_B_-siRNA with anti-tumor drug, cisplatin, was performed. Briefly, after the low concentration of ET_A_ or ET_B_-siRNA (20 nM) treatment, 2.5 μM of cisplatin that was a concentration slightly effective on cell growth inhibition was treated for 48 h. Cell growth was measured by Countess Automated Cell Counter and expressed as percentage.

### Statistical analysis

All results are expressed as mean ± SEM. Statistical comparisons were made using the Student-t test or Scheffe’s method after analysis of variances (ANOVA). The results were considered significantly different at P<0.05.

## Results

### Lingual SCCs in tumor tissues express ET_A_ and ET_B_

**Figure 1:**
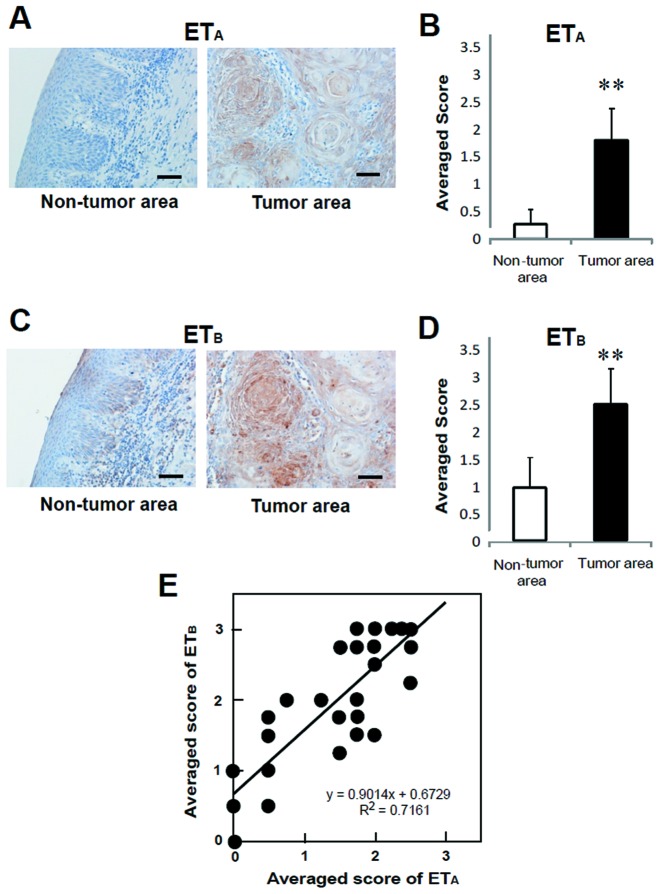
Expression of ET_A_ or ET_B_ on tumor cells in lingual tissues. (A) ET_A_ expression on tumor area (right panel) of primary lingual squamous cell carcinoma and non-tumor area (left panel) in the same tissue section (case 13 in Table I) by immunohistochemical observations. The brown color represents positive staining of ET_A_ and the blue represents counterstaining. Scale bar represents 100 μm. (B) Comparison of the expression of ET_A_ between tumor area and non-tumor area. Averaged score of strengthen of ET_A_ expression are expressed from the data in Table I. Each column represents mean ± SEM from 9–23 cases in tumor area or non-tumor area, respectively. ^**^P<0.01. (C) ET_B_ expression on tumor area (right panel) of primary lingual squamous cell carcinoma and non-tumor area (left panel) in the same tissue section (case 13 in Table I) by immunohistochemical observations. The brown color represents positive staining of ET_B_ and the blue represents counterstaining. Scale bar represents 100 μm. (D) Comparison of the expression of ET_B_ between tumor area and non-tumor area. Averaged score of strengthen of ET_B_ expression are expressed from the data in Table I. Each column represents mean ± SEM from 9–23 cases in tumor area or non-tumor area, respectively. ^**^P<0.01. (E) Correlation between ET_A_ and ET_B_ expression. Each point represents each individual.

Lingual SCC primary tissues were stained using anti-ET_A_ or anti-ET_B_ specific antibody, respectively. Positive staining of ET_A_ was observed in tumor area (Fig. 1A, right). In contrast, none of staining of ET_A_ was observed in non-tumor area in the same tissue section (Fig. 1A, left). Similar staining pattern was also observed in other tumor tissue sections (Table I). Statistically significance of the ET_A_ expression between tumor and non-tumor area was observed (Fig. 1B). In addition, positive staining of ET_B_ in tumor area, but not non-tumor area, was also observed in the same tissue section (Fig. 1C). Statistical significance of the ET_B_ expression between tumor and non-tumor areas was also observed (Fig. 1D and Table I). These results are similar to that of ET_A_. Good correlation between ET_A_ and ET_B_ expression was observed (Fig. 1E).

### ET receptor antagonists suppress cell growth of lingual and esophageal SCC

**Figure 2:**
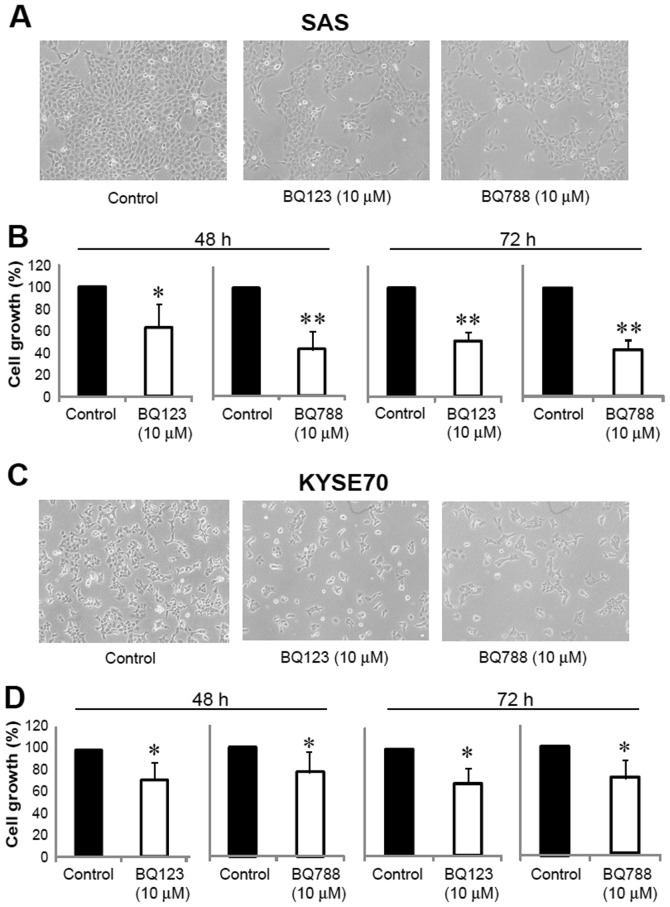
Effects of ET receptor antagonists on SCC cell growth. (A and B) Effect of ET_A_ antagonist, BQ123 or ET_B_ antagonist, BQ788 on cell growth of SAS. Typical images (A) and cell growth rate (B). 48 and 72 h after the start of treatment, viable cell number was counted and cell growth rate was expressed. Each column represents the percentage of cell growth (mean ± SEM from 4–5 independent experiments) compared to vehicle control (black column, PBS). Error bars represent standard deviations. ^**^P<0.01, ^*^P<0.05 vs. negative control. (C and D) Effect of ET_A_ antagonist, BQ123 or ET_B_ antagonist, BQ788 on cell growth of KYSE70.

According to the data of ET receptor expression in SCCs, we hypothesized that ET receptor-signalling might play an important role on the cell growth of SCCs. To investigate the hypothesis, we used ET receptor antagonists, BQ123 for ET_A_ and BQ788 for ET_B_. As shown in Fig. 2A and B, ET receptor antagonists, BQ123 and BQ788 suppressed the cell growth of lingual SCC cell line, SAS. The suppression by the antagonists was concentration- and time-dependent (data not shown).

In addition to the results of growth suppression of lingual SCC by the inhibition of ET receptors, both antagonists also suppressed the cell growth of esophageal SCC cell line, KYSE70 (Fig. 2C and D). These results indicate that ET receptor-signalling is required for the growth of SCCs.

### ET_A_ and ET_B_-siRNA suppress cell growth of lingual and esophageal SCC

**Figure 3:**
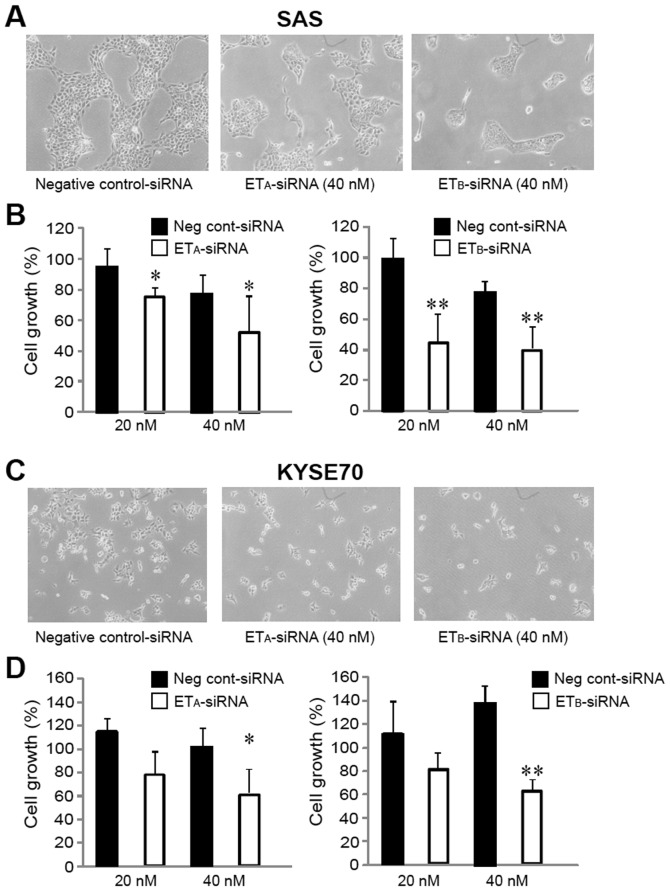
Effects of ET receptor knockdown on the cell growth of SCCs treated with ET_A_ or ET_B_-siRNA. (A and B) Effects of ET_A_-siRNA or ET_B_-siRNA on cell growth of SAS. Typical images (A) and cell growth rate (B). Cells were transfected the siRNA (40 nM) for 24 h and cultured for additional 48 h, followed by viable cell counting. Each value represents the percentage of cell growth compared with vehicle (non-siRNA) control from 4–7 independent experiments. White column represents the growth of cells transfected with ET_A_ (left panel) or ET_B_ (right panel)-siRNA and black column represents that of cells transfected with negative control-siRNA, respectively. Error bars represent standard deviations. ^**^P<0.01, ^*^P<0.05 vs. negative control. (C and D) Effects of ET_A_-siRNA and ET_B_-siRNA on cell growth of KYSE70. Typical photos (C) and cell growth rate (D). Cells were transfected the siRNA (40 nM) for 24 h and cultured for additional 48 h, followed by viable cell counting. Each value represents the percentage of cell growth compared with vehicle (non-siRNA) control from 4 independent experiments. White column represents the growth of cells transfected with ET_A_ (left panel) or ET_B_ (right panel)-siRNA and black column represents that of cells transfected with negative control-siRNA, respectively. Error bars represent standard deviations. ^**^P<0.01, ^*^P<0.05 vs. negative control.

To clarify the exact function of ET receptors on the growth of SCCs, we used small interfering RNA (siRNA) for ET_A_ and ET_B_. ET_A_ and ET_B_-siRNA effectively decreased the ET receptor protein levels in SCCs. The inhibition of cell growth on SAS was clearly observed when ET_A_ or ET_B_ was knocked down by the treatment with siRNA (Fig. 3A and B). Similar suppression of cell growth by the knockdown of ET_A_ or ET_B_ was also observed when esophageal SCC cell line, KYSE70 was treated with siRNA for ET_A_ or ET_B_ (Fig. 3C and D). These results clearly indicate that ET receptor-signalling is required for the growth of SCCs.

### Investigation of potential mechanisms

**Figure 4:**
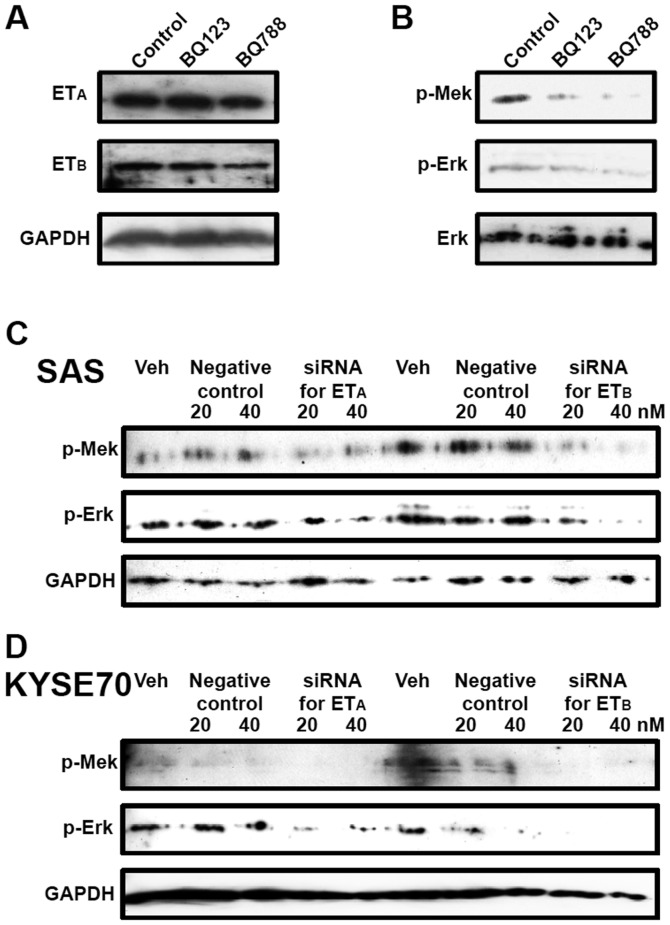
Involvement of MAPK pathway on ET receptor-signalling of SCC growth. (A) A Western blot analysis showing ET_A_ or ET_B_ expression in SAS which were pre-treated with ET_A_ antagonist, BQ123, or ET_B_ antagonist, BQ788. GAPDH is standard for equivalent application. No alteration was observed. (B) Suppression of MAPK pathway by ET receptor antagonists. SAS was treated with ET_A_ antagonist, BQ123, or ET_B_ antagonist, BQ788, then the phosphorylation of Mek (p-MEK) or Erk (p-Erk) was detected for Western blot analysis. (C) Western blot analysis for expression of p-MEK and p-Erk by the treatment with ET_A_ or ET_B_-siRNA on SAS. Cells were treated with ET_A_ or ET_B_-siRNA (20 and 40 nM), negative control siRNA and vehicle (Veh). Samples were collected for 24 h after the treatment. GAPDH was used to evaluate equivalent loading. (D) Western blot analysis for expression of p-MEK and p-Erk by the treatment with ET_A_ or ET_B_-siRNA on SAS. Cells were treated with ET_A_ or ET_B_-siRNA (20 and 40 nM), negative control siRNA and vehicle (Veh). Samples were collected 24 h after the treatment. GAPDH was used to evaluate equivalent loading.

We next investigated the mechanisms of inhibition of cell growth induced by the suppression of ET receptor-signalling. Western blot analysis showed the expression of ET_A_ and ET_B_ proteins on the lingual SCC cell line SAS (Fig. 4A). Although the specific antagonists blocked the ET_A_ or ET_B_ signalling, no alterations of receptor protein expression levels were observed (Fig. 4A). In contrast, blockade of ET receptor-signalling by the treatment with antagonists caused the suppression of phosphorylation of MEK and Erk (mitogen-activated protein kinase), the important members of MAPK pathway (Fig. 4B). In addition, similar suppression of MAPK pathway by knockdown of ET receptors was observed when SAS and KYSE70 were treated with ET_A_ or ET_B_-siRNA (Fig. 4C and D). These results indicate the involvement of MAPK pathway on the ET receptor-signalling mediated cell growth of SCCs.

In contrast, no inhibition of phosphorylation of focal adhesion kinase (FAK), a 125 kDa non-receptor tyrosine kinase (20,21), by the suppression of ET receptor signalling was observed (data not shown).

We also investigated the effect of blockade of ET receptor-signalling on expression of integrins such as integrin α5 and β1 (22,23). However, no alterations of integrin α5 and β1 expressions were observed (data not shown). These results suggest that the cell growth suppression of SCCs by the knockdown or blockade of ET receptors is mediated through the direct inhibition of MAPK signalling pathway.

### Combination therapy of ET_A_ or ET_B_-siRNA and anti-tumor drugs

**Figure 5:**
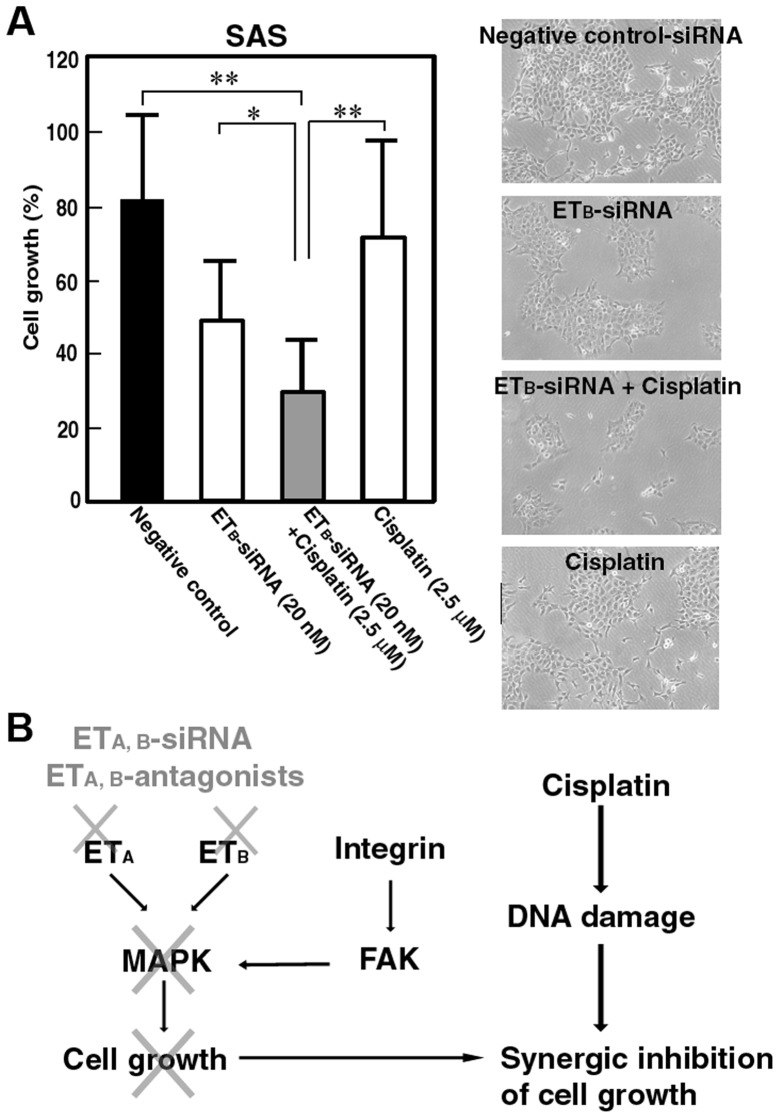
Combination therapy of ET_B_-siRNA and anti-tumor drug. (A) Combined treatment of low dose of ET_B_-siRNA (20 nM) with cisplatin (2.5 μM) was performed on cultured SCC cell line, SAS. Cells were treated with ET_B_-siRNA, negative control or vehicle for 24 h, then, treated with cisplatin or vehicle for 48 h. Each value represents the percentage of cell growth compared with vehicle (non-siRNA) control from 5 independent experiments. ^*^P<0.05, ^**^P<0.01 vs. negative control, or each single treatment, respectively. (B) Schematic illustration of possible mechanisms on the inhibition of cell growth by the combination of ET receptor-siRNA with cisplatin.

Reduction of dosage of anti-tumor drugs for cancer chemotherapy is clinically important to minimize the side effects, although the complete tumor cell death is required. Combined treatment of ET_B_-siRNA (20 nM) with anti-tumor drug, cisplatin (2.5 μM), drastically inhibited the cell growth of SAS in comparison to that in each single treatment (Fig. 5A). Similar results were also observed in the combined treatment of ET_A_-siRNA (20 nM) with cisplatin (data not shown). These results indicate that combination therapy of ET_A_ or ET_B_-siRNA and ordinal anti-tumor drugs may be a novel and useful therapy for SCCs.

## Discussion

There have been several reports on the expression of ET receptors in various human cancers (5–7), and it is considered to be the relationship between ET receptor-signalling and tumor cell growth. There are, however, few reports on the evaluation and investigation of the exact role of ET receptor-signalling using human SCC tissues and cultured cell lines of oral and esophageal carcinomas.

In the present study, using an immunohistochemical method, we demonstrated significantly higher levels of expression of ET_A_ and ET_B_ protein in human lingual cancer tissues than in non-tumor areas in the same tissue samples. Similar results were also observed on the cultured SCC cell lines such as SAS, lingual SCC, and KYSE70, esophageal SCC. These results indicate the involvement of ET receptor-signalling on SCC growth. Furthermore, we showed that the suppression of ET receptor protein by siRNA or the blockade by antagonists caused the inhibition of SCC growth. In our experimental conditions, both the treatment with ET_A_ and ET_B_ antagonists and siRNA strongly inhibited the cell growth of SCCs. These results strongly suggest the important role for ET receptor-signalling in SCC cell survival. In fact, recent reports strongly indicated the involvement of ET and its receptor on oral cancer (24,25). In addition, it was also reported that suppression of endothelin-converting enzyme-1 caused the inhibition of SCC proliferation (26). Our results, together with those reports, strongly suggest the importance of ET synthesis and its receptor-signalling pathway on oral SCC proliferation.

It is reported that phosphorylation of FAK is involved in the inhibition of apoptosis and promote cell growth in SCC cell lines (15,18). FAK is a 125 kDa non-receptor tyrosine kinase and an important regulator of cell survival, invasion, migration, and cell cycle progression (15,18,20,21). FAK is functionally important in transducing intracellular messages that are associated with growth factor signalling (15,18,20,21,27). The intracellular messages link p-FAK at Tyr^925^ to signalling pathways that activate MAPK cascades. In our present study, however, the inhibition of phosphorylation of FAK in SCCs treated with ET antagonists and siRNAs was not observed. In contrast, the inhibition of the phosphorylation of MEK and Erk by the treatment with ET antagonists and siRNAs was clearly observed. These results indicate that the inhibition of MAPK pathway by the suppression of ET receptor-signalling is due to the direct inhibition of MAPK pathway, but not through FAK pathway (Fig. 5B). Several reports have indicated the coupling of ET receptor-signalling and MAPK pathway (28,29). Our results agree with those reports and indicate that the mechanisms of the inhibition of cell growth by ET receptor-siRNAs and antagonists are, in part, due to the inhibition of MAPK pathway.

Reduction of dosage of anti-tumor drugs for cancer chemotherapy is clinically important to minimize the side effects, although the complete tumor cell death is required. Combined treatment of low concentration of ET receptor-siRNA (20 nM) with low concentration of anti-tumor drug, cisplatin (2.5 μM), drastically inhibited the cell growth of SAS in comparison to that in each single treatment. Cisplatin is extensively characterized as DNA damaging agent and the cytotoxicity of cisplatin is attributed to the ability to form inter and intra-strand nuclear DNA crosslinks (30,31). In contrast, inhibition of cell growth by ET receptor-siRNAs presented in our study was mainly due to the direct inhibition of MAPK pathway. Therefore, those two pathways on growth inhibition are different. This difference of mechanisms between ET receptor-siRNA and cisplatin may lead to show synergistic effect on the inhibition of tumor cell growth (Fig. 5A and B). Our results indicate that the decrease in ET receptor levels in SCCs that strongly express ET receptors increases the sensitivity against chemotherapy, and that the siRNA for ET receptors combined with anti-tumor drugs might be a useful therapy to reduce the dosage of anti-tumor drugs.

In summary, we showed the overexpression of ET_A_ and ET_B_ in tumor cells of human primary lingual SCC tissues and cultured SCC cell lines, and suggest a potentially important role for ET receptor-signalling on the cell growth of human SCCs.

## Figures and Tables

**Table I:** Profile of lingual squamous cell carcinoma patients, their histological diagnosis and expression of ET_A_ and ET_B_ in tissue sections.

Case	Age/Gender	Differentiation	ET_A_ expression		ET_B_ expression	
			Tumor area	Non-tumor area	Tumor area	Non-tumor area
1	71/F	Well differentiated SCC	+++	+	+++	++
2	57/M	Well differentiated SCC	+	N/A	++	N/A
3	69/M	Well differentiated SCC	++	N/A	+++	N/A
4	64/M	Well differentiated SCC	+++	+	+++	++
5	46/F	Well differentiated SCC	++	+	+++	+
6	61/M	Well differentiated SCC	+	N/A	+	N/A
7	48/M	Well differentiated SCC	++	N/A	+++	N/A
8	72/M	Well differentiated SCC	++	N/A	+++	N/A
9	79/M	Well differentiated SCC	++	N/A	++	N/A
10	68/M	Moderately differentiated SCC	+++	N/A	+++	N/A
11	64/M	Moderately differentiated SCC	++	+	+++	++
12	58/F	Moderately differentiated SCC	+++	N/A	+++	N/A
13	92/F	Moderately differentiated SCC	++	−	+++	−
14	86/F	Moderately differentiated SCC	++	+	+	+
15	57/M	Moderately differentiated SCC	+++	N/A	+++	N/A
16	62/M	Moderately differentiated SCC	++	N/A	+++	N/A
17	52/F	Poor-moderately differentiated SCC	++	N/A	++	N/A
18	38/M	Poorly differentiated SCC	+	−	++	+
19	51/F	Poorly differentiated SCC	++	−	++	+
20	67/M	Poorly differentiated SCC	++	N/A	++	N/A
21	66/M	Poorly differentiated SCC	++	N/A	+++	N/A
22	65/M	Poorly differentiated SCC	++	N/A	+++	N/A
23	33/M	Poorly differentiated SCC	++	−	+++	+

Expression of ET_A_ or ET_B_ by immunohistochemical staining in tumor and non-tumor area is scored and expressed as (−) to (+++). N/A, not applicable.
